# Dicyclo­hexyl­ammonium (*S*)-2-azido-3-phenyl­propano­ate

**DOI:** 10.1107/S1600536812025536

**Published:** 2012-06-13

**Authors:** Sebastian J. Petrik, Christopher L. Brown, Sue E. Boyd, Peter C. Healy

**Affiliations:** a Eskitis Institute for Cell and Molecular Therapies, Griffith University, Nathan, Brisbane 4111, Australia; b Queensland Micro and Nanotechnology Centre, Griffith University, Nathan, Brisbane 4111, Australia

## Abstract

The asymmetric unit of the title compound, C_12_H_24_N^+^·C_9_H_8_N_3_O_2_^−^, consists of two dicyclo­hexyl­ammonium cations linked to two (*S*)-2-azido-3-phenyl­propano­ate anions by four short N—H⋯O hydrogen bonds with N⋯O distances in the range 2.712 (3)–2.765 (3) Å. The dicyclo­hexyl­ammonium cations and the aryl and carboxyl­ate groups of the anion are related by a pseudo-inversion centre, with overall crystallographic inversion symmetry for the structure broken by the chirality of the α-C atoms of the anions.

## Related literature

For potential inhibitors of malarial proteases, see: Gardiner *et al.* (2009 ▶). For background to the synthesis, see: Goddard-Borger & Stick (2007 ▶). For related structures, see: Judaš & Portada (2008 ▶); Ng *et al.* (2001 ▶); Zain & Ng (2007 ▶). For graph-set analysis, see: Etter *et al.* (1990 ▶).
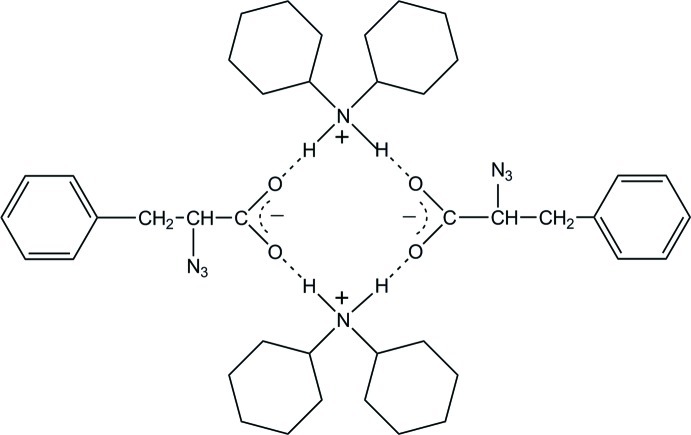


## Experimental

### 

#### Crystal data

C_12_H_24_N^+^·C_9_H_8_N_3_O_2_^−^
*M* *_r_* = 372.51
Triclinic, 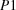

*a* = 9.4557 (7) Å
*b* = 11.0580 (6) Å
*c* = 11.0715 (8) Å
α = 113.187 (6)°
β = 99.919 (6)°
γ = 92.815 (5)°
*V* = 1039.46 (13) Å^3^
*Z* = 2
Mo *K*α radiation
μ = 0.08 mm^−1^
*T* = 200 K
0.48 × 0.41 × 0.37 mm

#### Data collection

Oxford Diffraction Gemini S Ultra diffractometer
Absorption correction: multi-scan (*CrysAlis PRO*; Agilent, 2012 ▶) *T*_min_ = 0.964, *T*_max_ = 0.972
6774 measured reflections
4986 independent reflections
4410 reflections with *I* > 2σ(*I*)
*R*_int_ = 0.024

#### Refinement

*R*[*F* ^2^ > 2σ(*F* ^2^)] = 0.042
*wR*(*F* ^2^) = 0.100
*S* = 1.06
4986 reflections
487 parameters
3 restraints
H-atom parameters constrained
Δρ_max_ = 0.25 e Å^−3^
Δρ_min_ = −0.15 e Å^−3^

### 

Data collection: *CrysAlis PRO* (Agilent, 2012 ▶); cell refinement: *CrysAlis PRO*; data reduction: *CrysAlis PRO*; program(s) used to solve structure: *SIR97* (Altomare *et al.*, 1999 ▶); program(s) used to refine structure: *TEXSAN* (Molecular Structure Corporation, 2001 ▶) and *SHELXL97* (Sheldrick, 2008 ▶); molecular graphics: *ORTEP-3 for Windows* (Farrugia, 1997 ▶); software used to prepare material for publication: *PLATON* (Spek, 2009 ▶) and *publCIF* (Westrip, 2010 ▶).

## Supplementary Material

Crystal structure: contains datablock(s) global, I. DOI: 10.1107/S1600536812025536/tk5110sup1.cif

Structure factors: contains datablock(s) I. DOI: 10.1107/S1600536812025536/tk5110Isup2.hkl

Supplementary material file. DOI: 10.1107/S1600536812025536/tk5110Isup3.cml

Additional supplementary materials:  crystallographic information; 3D view; checkCIF report

## Figures and Tables

**Table 1:** Hydrogen-bond geometry (Å, °)

*D*—H⋯*A*	*D*—H	H⋯*A*	*D*⋯*A*	*D*—H⋯*A*
N3—H3*A*⋯O11	0.85	1.93	2.765 (3)	168
N3—H3*B*⋯O22	0.85	1.88	2.712 (3)	167
N5—H5*A*⋯O12	0.85	1.90	2.741 (3)	168
N5—H5*B*⋯O21	0.85	1.90	2.725 (3)	164

**Fig. 1.**
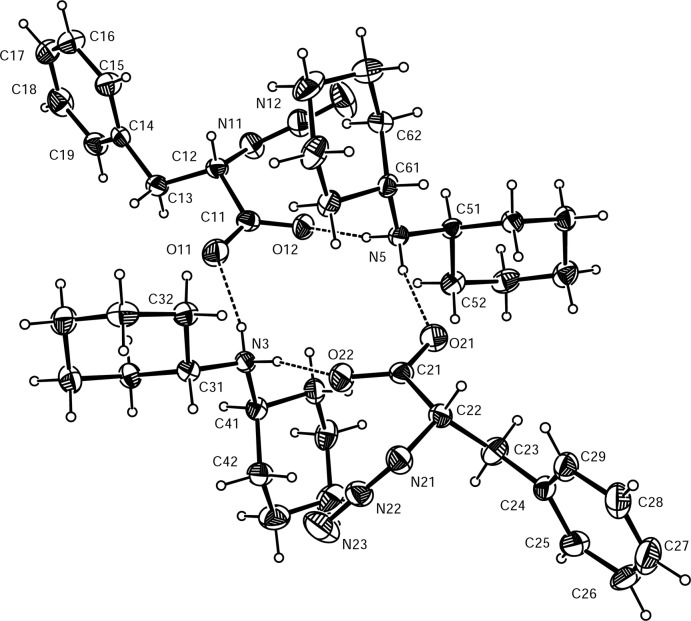
View of the molecules in the title compound with the atom numbering scheme. Displacement ellipsoids for non-H atoms are drawn at the 40% probability level. Hydrogen atoms are shown as spheres of arbitrary radius. Hydrogen bonds are shown as dashed lines.

## supplementary crystallographic information

### Comment

Recent investigations into the development of new molecules to act as potential inhibitors of malarial proteases (Gardiner *et al.*, 2009) resulted in the synthesis of the title compound (I) through utilization of the azido-transfer reagent imidazole-1-sulfonyl azide hydrochloride (Goddard-Borger & Stick, 2007). The structure of (I) is shown in Fig. 1 to consist of two dicyclohexylammonium cations and two (*S*)-2-azido-3-phenylpropanoate anions linked by four N—H···O hydrogen bonds with N···O = 2.712 (3) - 2.765 (3) Å, [graph set *R*_4_^4^(12); Etter *et al.*, 1990] (Table 1). The carboxylate C—O bond lengths span a narrow range from 1.247 (4) - 1.249 (4) Å. This structural format is similar to a number of other dimeric dicyclohexylammonium carboxylate salts (*e.g.* Ng *et al.*, 2001; Zain & Ng, 2007; Judaš & Portada, 2008) in which the ion-pairs are disposed about a crystallographic inversion centre. In the present structure, the Cy_2_NH_2_^+^ cations and the aryl and carboxylate groups of the anions are related by a pseudo-inversion centre, with overall crystallographic inversion symmetry for the structure broken by the chirality of the α-carbon of the anions.

### Experimental

(*L*)-phenylalanine (1.00 g, 6.05 mmol), imidazole-1-sulfonyl azide hydrochloride (1.52 g, 7.25 mmol), and copper sulfate pentahydrate (0.015 g, 0.06 mmol) were dissolved in methanol (30 ml) at 273 K. Anhydrous potassium carbonate (1.00 g, 7.24 mmol) was introduced over 5 minutes with stirring. The heterogeneous mixture was allowed to return to room temperature, and stirred for a further 16 h. Volatiles were removed *in vacuo* and the resulting material was suspended in water (90 ml). The mixture was acidifed to pH < 2 by dropwise addition of concentrated aqueous hydrochloric acid solution (37%). The resulting mixture was extracted with ethyl acetate (3 *x* 60 ml). The organic phases were combined, dried over anhydrous magnesium sulfate and volatiles were removed *in vacuo* to afford a crude oil. The crude oil was purified by flash chromatography (89:10:1 hexane:ethyl acetate:acetic acid) and the resulting oil dissolved in an excess of diethyl ether at 273 K. Dicyclohexylamine was added dropwise until pH > 10 was achieved by water-wet litmus paper, resulting in precipitation of (I). This was filtered, washed with excess cold diethyl ether and dried *in vacuo* to give (I) as an opaque white solid. Yield 0.91 g, 82%. Colourless crystals suitable for X-ray diffraction studies were grown by slow evaporation of an acetone/methanol solution of the compound.

^1^H NMR NMR (400 MHz, 298 K, d^6^-DMSO): δ (anion) 7.22 (m, 5H, Ar*H*), 3.64 (dd, J=9.3,4.5 Hz, 2H, C*H*), 3.06 (dd, J=14.0, 4.5 Hz, 2H, C*H_2_*)), 2.78 (dd, J=14.0, 9.3 Hz, 2H, C*H_2_*)). δ (cation) 2.99 (m, 2H, C*H*), 1.97 (m, 4H, C*H_2_*α), 1.72 (m, 4H, C*H_2_*β), 1.59 (m, 2H, C*H_2_*γ), 1.28 (m, 8H, C*H_2_*α,β), 1.08 (m, 2H, C*H_2_*γ). ^13^C NMR (100 MHz, d^6^-DMSO): δ 171.6, 139.0, 129.0, 128.0, 126.0, 65.4, 51.7, 37.9, 28.8, 24.9, 24.1. ESMS (-ve mode): carboxylate anion calcd 190.18, found 189.91.

### Refinement

The carbon-bound H atoms were constrained as riding atoms with C—H = 0.95 Å. The ammonium protons were located in difference Fourier maps and constrained with N—H 0.85 Å. *U*_iso_(H) values were set at 1.2*U*_eq_ of the parent atom. In the absence of significant anomalous scatterers in the compound, Friedel equivalents were merged with the absolute configuration assigned from the chirality of the *L*-phenylalanine precursor.

### Crystal data

**Table:**

C_12_H_24_N^+^·C_9_H_8_N_3_O_2_^−^	*Z* = 2
*M**_r_* = 372.51	*F*(000) = 404
Triclinic, *P*1	*D*_x_ = 1.190 Mg m^−^^3^
Hall symbol: P 1	Mo *K*α radiation, λ = 0.71070 Å
*a* = 9.4557 (7) Å	Cell parameters from 3545 reflections
*b* = 11.0580 (6) Å	θ = 3.2–30.5°
*c* = 11.0715 (8) Å	µ = 0.08 mm^−^^1^
α = 113.187 (6)°	*T* = 200 K
β = 99.919 (6)°	Block, colourless
γ = 92.815 (5)°	0.48 × 0.41 × 0.37 mm
*V* = 1039.46 (13) Å^3^	

### Data collection

**Table:**

Oxford Diffraction Gemini S Ultra diffractometer	4986 independent reflections
Radiation source: Enhance (Mo) X-ray Source	4410 reflections with *I* > 2σ(*I*)
Graphite monochromator	*R*_int_ = 0.024
Detector resolution: 16.0774 pixels mm^-1^	θ_max_ = 25.0°, θ_min_ = 3.2°
ω and φ scans	*h* = −11→10
Absorption correction: multi-scan (*CrysAlis PRO*; Agilent, 2012)	*k* = −13→13
*T*_min_ = 0.964, *T*_max_ = 0.972	*l* = −13→11
6774 measured reflections	

### Refinement

**Table:**

Refinement on *F*^2^	Primary atom site location: structure-invariant direct methods
Least-squares matrix: full	Secondary atom site location: difference Fourier map
*R*[*F*^2^ > 2σ(*F*^2^)] = 0.042	Hydrogen site location: inferred from neighbouring sites
*wR*(*F*^2^) = 0.100	H-atom parameters constrained
*S* = 1.06	*w* = 1/[σ^2^(*F*_o_^2^) + (0.0507*P*)^2^ + 0.0496*P*] where *P* = (*F*_o_^2^ + 2*F*_c_^2^)/3
4986 reflections	(Δ/σ)_max_ = 0.031
487 parameters	Δρ_max_ = 0.25 e Å^−^^3^
3 restraints	Δρ_min_ = −0.15 e Å^−^^3^

### Special details

**Table:**

Geometry. Bond distances, angles *etc*. have been calculated using the rounded fractional coordinates. All su's are estimated from the variances of the (full) variance-covariance matrix. The cell e.s.d.'s are taken into account in the estimation of distances, angles and torsion angles
Refinement. Refinement on *F*^2^ for ALL reflections except those flagged by the user for potential systematic errors. Weighted *R*-factors *wR* and all goodnesses of fit *S* are based on *F*^2^, conventional *R*-factors *R* are based on *F*, with *F* set to zero for negative *F*^2^. The observed criterion of *F*^2^ > σ(*F*^2^) is used only for calculating -*R*-factor-obs *etc*. and is not relevant to the choice of reflections for refinement. *R*-factors based on *F*^2^ are statistically about twice as large as those based on *F*, and *R*-factors based on ALL data will be even larger.

### Fractional atomic coordinates and isotropic or equivalent isotropic displacement parameters (Å^2^)

**Table:**

	*x*	*y*	*z*	*U*_iso_*/*U*_eq_	
N3	0.6721 (2)	0.4822 (2)	0.3905 (2)	0.0270 (7)	
C31	0.7523 (3)	0.5718 (2)	0.3451 (3)	0.0277 (9)	
C32	0.7019 (3)	0.7080 (3)	0.4040 (3)	0.0336 (10)	
C33	0.7875 (4)	0.8105 (3)	0.3743 (3)	0.0415 (10)	
C34	0.9497 (4)	0.8148 (3)	0.4160 (3)	0.0449 (11)	
C35	0.9940 (3)	0.6778 (3)	0.3502 (3)	0.0399 (11)	
C36	0.9146 (3)	0.5778 (3)	0.3869 (3)	0.0324 (9)	
C41	0.7050 (3)	0.3416 (2)	0.3482 (3)	0.0292 (9)	
C42	0.6864 (3)	0.2703 (3)	0.1971 (3)	0.0395 (10)	
C43	0.7139 (4)	0.1261 (3)	0.1581 (4)	0.0526 (13)	
C44	0.6181 (4)	0.0541 (3)	0.2114 (4)	0.0551 (11)	
C45	0.6374 (3)	0.1266 (3)	0.3627 (4)	0.0442 (11)	
C46	0.6080 (3)	0.2699 (3)	0.4024 (3)	0.0364 (10)	
N5	0.3324 (2)	0.5072 (2)	0.6097 (2)	0.0274 (7)	
C51	0.2476 (3)	0.4200 (2)	0.6542 (3)	0.0274 (8)	
C52	0.2925 (3)	0.2812 (3)	0.5961 (3)	0.0354 (9)	
C53	0.2047 (4)	0.1833 (3)	0.6287 (3)	0.0440 (11)	
C54	0.0424 (4)	0.1825 (3)	0.5865 (3)	0.0440 (11)	
C55	0.0014 (3)	0.3215 (3)	0.6486 (3)	0.0375 (10)	
C56	0.0861 (3)	0.4171 (3)	0.6102 (3)	0.0311 (9)	
C61	0.3032 (3)	0.6489 (2)	0.6513 (3)	0.0290 (9)	
C62	0.3270 (3)	0.7228 (3)	0.8025 (3)	0.0369 (10)	
C63	0.2998 (4)	0.8665 (3)	0.8391 (4)	0.0532 (11)	
C64	0.3947 (4)	0.9353 (3)	0.7823 (4)	0.0567 (13)	
C65	0.3739 (4)	0.8604 (3)	0.6302 (4)	0.0487 (11)	
C66	0.3998 (3)	0.7171 (3)	0.5926 (3)	0.0364 (10)	
O11	0.7677 (2)	0.6136 (2)	0.6656 (2)	0.0491 (8)	
O12	0.6097 (2)	0.52022 (19)	0.7418 (2)	0.0388 (7)	
N11	0.7730 (3)	0.6229 (3)	0.9980 (3)	0.0425 (9)	
N12	0.6464 (3)	0.6260 (3)	1.0145 (3)	0.0455 (10)	
N13	0.5370 (4)	0.6264 (4)	1.0423 (4)	0.0715 (14)	
C11	0.7210 (3)	0.5944 (3)	0.7561 (3)	0.0311 (9)	
C12	0.8073 (3)	0.6765 (2)	0.9020 (3)	0.0319 (8)	
C13	0.9694 (3)	0.6833 (3)	0.9105 (3)	0.0355 (9)	
C14	1.0640 (3)	0.7758 (3)	1.0441 (3)	0.0305 (9)	
C15	1.0415 (3)	0.9078 (3)	1.1071 (3)	0.0401 (10)	
C16	1.1350 (4)	0.9951 (3)	1.2242 (3)	0.0485 (11)	
C17	1.2510 (4)	0.9514 (3)	1.2807 (3)	0.0499 (11)	
C18	1.2741 (4)	0.8212 (4)	1.2207 (4)	0.0511 (11)	
C19	1.1817 (3)	0.7334 (3)	1.1023 (3)	0.0381 (10)	
O21	0.2250 (2)	0.3996 (2)	0.3395 (2)	0.0471 (8)	
O22	0.3981 (2)	0.4656 (2)	0.2568 (2)	0.0441 (7)	
N21	0.2214 (3)	0.4049 (2)	0.0103 (3)	0.0397 (8)	
N22	0.3342 (3)	0.3547 (3)	−0.0129 (3)	0.0413 (9)	
N23	0.4348 (4)	0.3164 (3)	−0.0464 (3)	0.0681 (11)	
C21	0.2724 (3)	0.4189 (3)	0.2494 (3)	0.0314 (9)	
C22	0.1623 (3)	0.3814 (3)	0.1162 (3)	0.0324 (9)	
C23	0.0814 (3)	0.2439 (3)	0.0665 (3)	0.0396 (10)	
C24	−0.0451 (3)	0.2037 (3)	−0.0512 (3)	0.0346 (9)	
C25	−0.0806 (4)	0.0706 (3)	−0.1401 (3)	0.0511 (11)	
C26	−0.2041 (5)	0.0315 (4)	−0.2444 (4)	0.0657 (14)	
C27	−0.2891 (4)	0.1231 (4)	−0.2599 (4)	0.0645 (14)	
C28	−0.2552 (4)	0.2537 (4)	−0.1757 (3)	0.0520 (11)	
C29	−0.1342 (3)	0.2933 (3)	−0.0713 (3)	0.0393 (10)	
H3A	0.69010	0.51810	0.47570	0.0330*	
H3B	0.58240	0.47940	0.36060	0.0330*	
H31	0.72700	0.53870	0.24980	0.0330*	
H32A	0.60230	0.70160	0.36600	0.0400*	
H32B	0.71470	0.73700	0.49850	0.0400*	
H33A	0.76140	0.89600	0.42160	0.0500*	
H33B	0.76390	0.78870	0.28040	0.0500*	
H34A	0.99840	0.87490	0.38900	0.0540*	
H34B	0.97600	0.84550	0.51080	0.0540*	
H35A	1.09550	0.68230	0.37950	0.0480*	
H35B	0.97170	0.65020	0.25540	0.0480*	
H36A	0.94250	0.49320	0.34190	0.0390*	
H36B	0.94040	0.60430	0.48110	0.0390*	
H41	0.80300	0.34380	0.38800	0.0350*	
H42A	0.75380	0.31410	0.16750	0.0480*	
H42B	0.59100	0.27300	0.15530	0.0480*	
H43A	0.69580	0.08220	0.06310	0.0630*	
H43B	0.81250	0.12480	0.19430	0.0630*	
H44A	0.64290	−0.03280	0.18970	0.0670*	
H44B	0.52010	0.05000	0.17020	0.0670*	
H45A	0.57240	0.08310	0.39310	0.0530*	
H45B	0.73440	0.12610	0.40370	0.0530*	
H46A	0.62640	0.31460	0.49720	0.0440*	
H46B	0.50980	0.27140	0.36590	0.0440*	
H5A	0.42170	0.50900	0.64020	0.0330*	
H5B	0.31560	0.47200	0.52410	0.0330*	
H51	0.27170	0.45450	0.74940	0.0330*	
H52A	0.39210	0.28580	0.63230	0.0430*	
H52B	0.27790	0.25140	0.50110	0.0430*	
H53A	0.22910	0.09720	0.58270	0.0530*	
H53B	0.22900	0.20760	0.72280	0.0530*	
H54A	−0.00830	0.12670	0.61540	0.0530*	
H54B	0.01670	0.15090	0.49140	0.0530*	
H55A	−0.09900	0.32000	0.61790	0.0460*	
H55B	0.02260	0.35230	0.74370	0.0460*	
H56A	0.06050	0.50420	0.65210	0.0370*	
H56B	0.06240	0.38940	0.51520	0.0370*	
H61	0.20530	0.64930	0.61340	0.0350*	
H62A	0.26200	0.68180	0.83520	0.0440*	
H62B	0.42380	0.72130	0.84200	0.0440*	
H63A	0.32000	0.91260	0.93380	0.0640*	
H63B	0.20140	0.86820	0.80360	0.0640*	
H64A	0.37060	1.02300	0.80210	0.0670*	
H64B	0.49340	0.94050	0.82230	0.0670*	
H65A	0.44000	0.90260	0.59850	0.0590*	
H65B	0.27760	0.86320	0.58940	0.0590*	
H66A	0.37850	0.67190	0.49710	0.0440*	
H66B	0.49850	0.71490	0.62600	0.0440*	
H12	0.78290	0.76470	0.93010	0.0380*	
H13A	0.99050	0.71180	0.84380	0.0430*	
H13B	0.99480	0.59700	0.89110	0.0430*	
H15	0.96030	0.93860	1.06900	0.0480*	
H16	1.11870	1.08700	1.26500	0.0600*	
H17	1.31530	1.01100	1.36080	0.0610*	
H18	1.35440	0.79130	1.26040	0.0610*	
H19	1.20010	0.64450	1.06090	0.0460*	
H22	0.09160	0.44040	0.13710	0.0390*	
H23A	0.04570	0.23810	0.13900	0.0470*	
H23B	0.14870	0.18250	0.04070	0.0470*	
H25	−0.02210	0.00700	−0.13020	0.0610*	
H26	−0.22660	−0.05920	−0.30500	0.0790*	
H27	−0.37260	0.09500	−0.33020	0.0780*	
H28	−0.31350	0.31720	−0.18850	0.0630*	
H29	−0.11140	0.38480	−0.01220	0.0470*	

### Atomic displacement parameters (Å^2^)

**Table:**

	*U*^11^	*U*^22^	*U*^33^	*U*^12^	*U*^13^	*U*^23^
N3	0.0237 (13)	0.0331 (12)	0.0233 (12)	0.0025 (9)	0.0043 (10)	0.0110 (9)
C31	0.0284 (16)	0.0303 (14)	0.0259 (15)	0.0057 (11)	0.0044 (12)	0.0134 (11)
C32	0.0329 (18)	0.0336 (15)	0.0323 (17)	0.0068 (12)	0.0052 (13)	0.0119 (12)
C33	0.059 (2)	0.0333 (16)	0.0360 (18)	0.0120 (14)	0.0115 (15)	0.0168 (13)
C34	0.054 (2)	0.0369 (17)	0.045 (2)	−0.0037 (14)	0.0142 (16)	0.0176 (14)
C35	0.0359 (18)	0.0426 (17)	0.047 (2)	0.0028 (13)	0.0146 (15)	0.0223 (15)
C36	0.0290 (17)	0.0353 (15)	0.0366 (17)	0.0059 (11)	0.0081 (13)	0.0178 (12)
C41	0.0255 (16)	0.0281 (14)	0.0343 (16)	0.0063 (11)	0.0046 (12)	0.0135 (12)
C42	0.0438 (19)	0.0365 (16)	0.0362 (18)	0.0057 (13)	0.0121 (14)	0.0113 (13)
C43	0.065 (3)	0.0351 (17)	0.052 (2)	0.0122 (15)	0.0177 (18)	0.0091 (15)
C44	0.061 (2)	0.0345 (17)	0.062 (2)	0.0024 (15)	0.0063 (19)	0.0148 (16)
C45	0.040 (2)	0.0352 (16)	0.060 (2)	−0.0022 (13)	0.0048 (16)	0.0254 (15)
C46	0.0305 (18)	0.0421 (16)	0.0374 (18)	−0.0009 (12)	0.0064 (13)	0.0181 (13)
N5	0.0223 (13)	0.0294 (12)	0.0256 (13)	0.0032 (9)	0.0025 (10)	0.0072 (9)
C51	0.0305 (16)	0.0278 (14)	0.0223 (14)	0.0010 (11)	0.0044 (12)	0.0094 (11)
C52	0.0336 (17)	0.0315 (14)	0.0377 (18)	0.0078 (12)	0.0008 (14)	0.0128 (12)
C53	0.060 (2)	0.0287 (16)	0.0411 (19)	0.0034 (14)	0.0078 (16)	0.0133 (13)
C54	0.051 (2)	0.0375 (17)	0.043 (2)	−0.0063 (14)	0.0086 (16)	0.0179 (14)
C55	0.0343 (18)	0.0430 (17)	0.0341 (17)	−0.0029 (12)	0.0074 (13)	0.0155 (13)
C56	0.0285 (17)	0.0341 (15)	0.0303 (16)	0.0048 (12)	0.0071 (13)	0.0123 (12)
C61	0.0229 (15)	0.0276 (14)	0.0329 (16)	0.0014 (10)	0.0018 (12)	0.0104 (11)
C62	0.0392 (19)	0.0336 (15)	0.0347 (17)	0.0061 (12)	0.0116 (14)	0.0090 (12)
C63	0.061 (2)	0.0354 (17)	0.052 (2)	0.0081 (15)	0.0157 (18)	0.0044 (15)
C64	0.066 (3)	0.0242 (16)	0.064 (2)	−0.0044 (15)	0.003 (2)	0.0070 (15)
C65	0.052 (2)	0.0368 (17)	0.056 (2)	−0.0075 (14)	0.0006 (17)	0.0234 (15)
C66	0.0337 (18)	0.0362 (16)	0.0357 (17)	−0.0028 (12)	0.0026 (13)	0.0137 (13)
O11	0.0470 (15)	0.0621 (14)	0.0296 (13)	−0.0084 (10)	0.0006 (10)	0.0146 (10)
O12	0.0277 (12)	0.0475 (11)	0.0348 (12)	−0.0011 (9)	0.0002 (9)	0.0135 (9)
N11	0.0335 (16)	0.0633 (16)	0.0313 (14)	0.0057 (11)	0.0038 (12)	0.0214 (12)
N12	0.0406 (18)	0.0622 (17)	0.0338 (15)	−0.0010 (12)	0.0054 (13)	0.0216 (12)
N13	0.046 (2)	0.119 (3)	0.066 (2)	0.0054 (17)	0.0227 (16)	0.051 (2)
C11	0.0289 (17)	0.0361 (15)	0.0250 (16)	0.0086 (12)	0.0033 (13)	0.0094 (12)
C12	0.0291 (15)	0.0352 (14)	0.0267 (14)	0.0051 (11)	0.0053 (11)	0.0079 (11)
C13	0.0290 (15)	0.0387 (15)	0.0314 (15)	0.0051 (11)	0.0064 (12)	0.0065 (12)
C14	0.0272 (15)	0.0356 (15)	0.0283 (15)	0.0015 (11)	0.0104 (12)	0.0111 (12)
C15	0.0409 (19)	0.0384 (16)	0.0367 (17)	0.0076 (13)	0.0053 (14)	0.0119 (13)
C16	0.054 (2)	0.0409 (17)	0.0399 (18)	0.0009 (14)	0.0090 (16)	0.0064 (13)
C17	0.042 (2)	0.054 (2)	0.0346 (18)	−0.0097 (15)	0.0011 (15)	0.0028 (15)
C18	0.0332 (19)	0.072 (2)	0.049 (2)	0.0100 (16)	0.0033 (15)	0.0276 (17)
C19	0.0335 (18)	0.0418 (16)	0.0377 (17)	0.0096 (13)	0.0079 (14)	0.0141 (13)
O21	0.0435 (14)	0.0676 (14)	0.0264 (12)	−0.0018 (10)	0.0035 (10)	0.0178 (10)
O22	0.0272 (12)	0.0612 (13)	0.0365 (13)	0.0024 (9)	−0.0020 (9)	0.0161 (10)
N21	0.0338 (15)	0.0503 (14)	0.0363 (14)	0.0057 (11)	0.0060 (11)	0.0195 (11)
N22	0.0362 (16)	0.0554 (16)	0.0290 (14)	0.0072 (12)	0.0063 (12)	0.0140 (11)
N23	0.052 (2)	0.107 (2)	0.0491 (19)	0.0277 (18)	0.0226 (15)	0.0289 (17)
C21	0.0283 (17)	0.0319 (14)	0.0288 (16)	0.0083 (11)	0.0049 (13)	0.0068 (12)
C22	0.0266 (16)	0.0398 (15)	0.0281 (14)	0.0046 (11)	0.0049 (11)	0.0114 (12)
C23	0.0413 (18)	0.0367 (15)	0.0386 (17)	0.0017 (12)	0.0013 (14)	0.0164 (13)
C24	0.0322 (17)	0.0389 (16)	0.0279 (16)	−0.0076 (12)	0.0060 (12)	0.0102 (12)
C25	0.057 (2)	0.0448 (18)	0.040 (2)	−0.0065 (15)	0.0159 (17)	0.0045 (15)
C26	0.074 (3)	0.058 (2)	0.0340 (19)	−0.031 (2)	0.0090 (19)	−0.0081 (16)
C27	0.043 (2)	0.094 (3)	0.046 (2)	−0.023 (2)	−0.0047 (17)	0.027 (2)
C28	0.0344 (19)	0.080 (2)	0.0446 (19)	−0.0069 (16)	0.0025 (15)	0.0325 (18)
C29	0.0322 (17)	0.0500 (17)	0.0338 (16)	−0.0036 (13)	0.0020 (13)	0.0181 (13)

### Geometric parameters (Å, º)

**Table:**

O11—C11	1.247 (4)	C62—C63	1.525 (5)
O12—C11	1.249 (4)	C63—C64	1.511 (5)
O21—C21	1.247 (4)	C64—C65	1.527 (6)
O22—C21	1.249 (4)	C65—C66	1.516 (5)
N3—C41	1.503 (4)	C51—H51	0.9500
N3—C31	1.503 (4)	C52—H52B	0.9500
N3—H3A	0.8500	C52—H52A	0.9500
N3—H3B	0.8500	C53—H53B	0.9500
N5—C61	1.504 (4)	C53—H53A	0.9500
N5—C51	1.500 (4)	C54—H54B	0.9500
N5—H5A	0.8500	C54—H54A	0.9400
N5—H5B	0.8500	C55—H55A	0.9500
N11—C12	1.477 (4)	C55—H55B	0.9500
N11—N12	1.242 (4)	C56—H56B	0.9500
N12—N13	1.129 (5)	C56—H56A	0.9500
N21—N22	1.246 (4)	C61—H61	0.9500
N21—C22	1.485 (4)	C62—H62A	0.9400
N22—N23	1.122 (5)	C62—H62B	0.9500
C31—C32	1.525 (4)	C63—H63A	0.9500
C31—C36	1.515 (4)	C63—H63B	0.9500
C32—C33	1.531 (5)	C64—H64A	0.9500
C33—C34	1.516 (5)	C64—H64B	0.9500
C34—C35	1.517 (5)	C65—H65A	0.9500
C35—C36	1.526 (5)	C65—H65B	0.9500
C41—C46	1.524 (4)	C66—H66B	0.9500
C41—C42	1.516 (4)	C66—H66A	0.9500
C42—C43	1.528 (5)	C11—C12	1.555 (4)
C43—C44	1.512 (5)	C12—C13	1.516 (4)
C44—C45	1.518 (6)	C13—C14	1.516 (4)
C45—C46	1.523 (5)	C14—C15	1.394 (5)
C31—H31	0.9500	C14—C19	1.384 (4)
C32—H32B	0.9500	C15—C16	1.389 (4)
C32—H32A	0.9500	C16—C17	1.372 (5)
C33—H33A	0.9500	C17—C18	1.375 (6)
C33—H33B	0.9500	C18—C19	1.394 (5)
C34—H34B	0.9500	C12—H12	0.9500
C34—H34A	0.9600	C13—H13B	0.9500
C35—H35A	0.9500	C13—H13A	0.9600
C35—H35B	0.9500	C15—H15	0.9500
C36—H36A	0.9500	C16—H16	0.9700
C36—H36B	0.9500	C17—H17	0.9500
C41—H41	0.9500	C18—H18	0.9500
C42—H42A	0.9600	C19—H19	0.9500
C42—H42B	0.9500	C21—C22	1.542 (4)
C43—H43B	0.9500	C22—C23	1.511 (5)
C43—H43A	0.9500	C23—C24	1.510 (4)
C44—H44B	0.9500	C24—C25	1.395 (5)
C44—H44A	0.9500	C24—C29	1.390 (5)
C45—H45A	0.9400	C25—C26	1.406 (6)
C45—H45B	0.9500	C26—C27	1.368 (7)
C46—H46A	0.9500	C27—C28	1.360 (6)
C46—H46B	0.9500	C28—C29	1.391 (4)
C51—C52	1.527 (4)	C22—H22	0.9500
C51—C56	1.516 (4)	C23—H23A	0.9500
C52—C53	1.521 (5)	C23—H23B	0.9500
C53—C54	1.524 (5)	C25—H25	0.9400
C54—C55	1.518 (5)	C26—H26	0.9500
C55—C56	1.526 (5)	C27—H27	0.9500
C61—C66	1.532 (4)	C28—H28	0.9500
C61—C62	1.515 (4)	C29—H29	0.9500
			
O11···C32	3.419 (4)	H15···H12	2.3100
O11···N3	2.765 (3)	H17···H45A^vii^	2.4400
O11···C31	3.369 (4)	H17···H53A^vii^	2.5600
O12···N5	2.741 (3)	H17···H52B^vii^	2.5800
O12···C51	3.395 (3)	H19···H13B	2.3300
O12···N11	2.739 (4)	H19···N21^i^	2.5100
O12···N12	2.724 (4)	H22···C35^ii^	3.0700
O21···C56	3.416 (4)	H22···H29	2.1900
O21···N5	2.725 (3)	H22···C29	2.7400
O21···C51	3.370 (4)	H23A···O21	2.5100
O21···C61	3.390 (4)	H23B···H25	2.3900
O22···N3	2.712 (3)	H23B···N22	2.8200
O22···N21	2.751 (4)	H25···H23B	2.3900
O22···C31	3.337 (3)	H26···H32B^viii^	2.3900
O22···N23	3.195 (4)	H27···H65A^viii^	2.4700
O22···N22	2.685 (4)	H28···O12^iv^	2.7400
O11···H13A	2.4800	H29···N11^iv^	2.8700
O11···H32B	2.7000	H29···C22	2.7400
O11···H66B	2.8700	H29···H22	2.1900
O11···H36B	2.8000	H31···H35B	2.5500
O11···H3A	1.9300	H31···H42A	2.3300
O12···H5A	1.9000	H31···C42	2.7800
O12···H46A	2.8000	H32A···H3B	2.4300
O12···H28^i^	2.7400	H32A···O22	2.8700
O12···H62B	2.8900	H32B···H26^vii^	2.3900
O21···H36A^ii^	2.9100	H32B···O11	2.7000
O21···H23A	2.5100	H32B···H3A	2.3300
O21···H52B	2.8700	H33B···N12^v^	2.7600
O21···H56B	2.7100	H33B···H35B	2.5400
O21···H5B	1.9000	H34A···C17^v^	3.0700
O22···H3B	1.8800	H34A···C16^v^	3.0500
O22···H32A	2.8700	H34B···H36B	2.5500
O22···H42B	2.8900	H35B···H31	2.5500
O22···H66A	2.7800	H35B···H33B	2.5400
N3···O22	2.712 (3)	H36A···O21^vi^	2.9100
N3···O11	2.765 (3)	H36A···C41	2.7600
N5···O21	2.725 (3)	H36A···H41	2.3300
N5···O12	2.741 (3)	H36A···C42	3.0800
N11···O12	2.739 (4)	H36A···H42A	2.5100
N12···O12	2.724 (4)	H36B···H3A	2.4900
N13···N23^iii^	3.217 (6)	H36B···O11	2.8000
N13···N22^iii^	3.265 (6)	H36B···H61^vi^	2.5900
N21···C29	3.364 (4)	H36B···H34B	2.5500
N21···O22	2.751 (4)	H41···H36A	2.3300
N21···C19^iv^	3.424 (4)	H41···H43B	2.5400
N22···N13^v^	3.265 (6)	H41···H45B	2.5400
N22···O22	2.685 (4)	H41···C36	2.7500
N23···O22	3.195 (4)	H41···H56B^vi^	2.5300
N23···N13^v^	3.217 (6)	H42A···H31	2.3300
N11···H29^i^	2.8700	H42A···C29^vi^	2.9500
N12···H33B^iii^	2.7600	H42A···C31	2.7500
N13···H62B	2.8800	H42A···H36A	2.5100
N21···H19^iv^	2.5100	H42A···C36	3.0700
N22···H23B	2.8200	H42B···O22	2.8900
N22···H53B^v^	2.7000	H42B···N23	2.6800
N23···H53B^v^	2.7200	H42B···H3B	2.5300
N23···H42B	2.6800	H42B···H44B	2.6000
C19···N21^i^	3.424 (4)	H42B···H46B	2.5800
C28···C55^v^	3.582 (5)	H43B···H41	2.5400
C29···N21	3.364 (4)	H43B···H45B	2.5500
C31···O22	3.337 (3)	H44B···H42B	2.6000
C31···O11	3.369 (4)	H44B···H46B	2.5700
C32···O11	3.419 (4)	H45A···H17^viii^	2.4400
C36···C42	3.567 (5)	H45B···H43B	2.5500
C42···C36	3.567 (5)	H45B···H41	2.5400
C51···O12	3.395 (3)	H46A···O12	2.8000
C51···O21	3.370 (4)	H46A···H3A	2.4100
C55···C28^iii^	3.582 (5)	H46B···H3B	2.3900
C56···O21	3.416 (4)	H46B···H44B	2.5700
C61···O21	3.390 (4)	H46B···H42B	2.5800
C11···H66B	3.0000	H51···H55B	2.5400
C11···H3A	2.8300	H51···H62A	2.3200
C11···H5A	2.8400	H51···C62	2.7900
C12···H15	2.8900	H52A···H5A	2.4400
C13···H62A^vi^	3.0200	H52B···H5B	2.3500
C13···H56A^vi^	3.0700	H52B···O21	2.8700
C15···H12	2.8000	H52B···H56B	2.5900
C16···H34A^iii^	3.0500	H52B···H17^viii^	2.5800
C17···H34A^iii^	3.0700	H53A···H17^viii^	2.5600
C19···H62A^vi^	3.0200	H53B···N23^iii^	2.7200
C21···H3B	2.9100	H53B···N22^iii^	2.7000
C21···H66A	3.0100	H53B···H55B	2.5700
C21···H5B	2.8100	H54A···C26^iii^	3.0100
C22···H29	2.7400	H54B···H56B	2.5500
C26···H54A^v^	3.0100	H55B···H53B	2.5700
C29···H42A^ii^	2.9500	H55B···C29^iii^	2.9700
C29···H22	2.7400	H55B···H51	2.5400
C29···H55B^v^	2.9700	H56A···C13^ii^	3.0700
C31···H42A	2.7500	H56A···C61	2.7400
C35···H22^vi^	3.0700	H56A···H61	2.2700
C36···H42A	3.0700	H56B···O21	2.7100
C36···H41	2.7500	H56B···H54B	2.5500
C41···H36A	2.7600	H56B···H52B	2.5900
C42···H31	2.7800	H56B···H41^ii^	2.5300
C42···H36A	3.0800	H56B···H5B	2.4900
C51···H62A	2.7800	H61···H63B	2.5100
C56···H61	2.7400	H61···H56A	2.2700
C61···H56A	2.7400	H61···H36B^ii^	2.5900
C62···H51	2.7900	H61···H65B	2.5600
H3A···H46A	2.4100	H61···C56	2.7400
H3A···H36B	2.4900	H62A···C13^ii^	3.0200
H3A···O11	1.9300	H62A···C19^ii^	3.0200
H3A···C11	2.8300	H62A···C51	2.7800
H3A···H32B	2.3300	H62A···H51	2.3200
H3B···H46B	2.3900	H62B···H66B	2.5800
H3B···C21	2.9100	H62B···H5A	2.5200
H3B···H32A	2.4300	H62B···H64B	2.5800
H3B···O22	1.8800	H62B···O12	2.8900
H3B···H42B	2.5300	H62B···N13	2.8800
H5A···H52A	2.4400	H63B···H61	2.5100
H5A···H62B	2.5200	H63B···H65B	2.5800
H5A···C11	2.8400	H64B···H66B	2.5900
H5A···O12	1.9000	H64B···H62B	2.5800
H5A···H66B	2.4300	H65A···H27^vii^	2.4700
H5B···H66A	2.4000	H65B···H63B	2.5800
H5B···O21	1.9000	H65B···H61	2.5600
H5B···C21	2.8100	H66A···O22	2.7800
H5B···H52B	2.3500	H66A···C21	3.0100
H5B···H56B	2.4900	H66A···H5B	2.4000
H12···C15	2.8000	H66B···H64B	2.5900
H12···H15	2.3100	H66B···H5A	2.4300
H13A···O11	2.4800	H66B···H62B	2.5800
H13B···H19	2.3300	H66B···O11	2.8700
H15···C12	2.8900	H66B···C11	3.0000
			
C31—N3—C41	118.1 (2)	H53A—C53—H53B	110.00
C41—N3—H3A	108.00	C52—C53—H53A	109.00
C31—N3—H3A	107.00	C54—C53—H53A	109.00
C31—N3—H3B	107.00	C54—C53—H53B	109.00
C41—N3—H3B	107.00	H54A—C54—H54B	110.00
H3A—N3—H3B	110.00	C55—C54—H54A	109.00
C51—N5—C61	117.9 (2)	C55—C54—H54B	109.00
C51—N5—H5B	108.00	C53—C54—H54A	109.00
C51—N5—H5A	108.00	C53—C54—H54B	109.00
C61—N5—H5B	107.00	C56—C55—H55A	109.00
H5A—N5—H5B	109.00	C54—C55—H55B	109.00
C61—N5—H5A	107.00	C54—C55—H55A	109.00
N12—N11—C12	115.4 (3)	H55A—C55—H55B	110.00
N11—N12—N13	172.1 (4)	C56—C55—H55B	109.00
N22—N21—C22	114.4 (3)	C51—C56—H56B	109.00
N21—N22—N23	170.7 (4)	H56A—C56—H56B	109.00
N3—C31—C36	111.2 (2)	C51—C56—H56A	109.00
C32—C31—C36	112.1 (2)	C55—C56—H56A	110.00
N3—C31—C32	107.5 (2)	C55—C56—H56B	109.00
C31—C32—C33	111.6 (2)	C66—C61—H61	108.00
C32—C33—C34	112.3 (3)	C62—C61—H61	108.00
C33—C34—C35	110.2 (3)	N5—C61—H61	108.00
C34—C35—C36	111.1 (3)	C63—C62—H62A	109.00
C31—C36—C35	110.5 (3)	C61—C62—H62A	109.00
N3—C41—C42	112.2 (2)	C61—C62—H62B	109.00
N3—C41—C46	108.4 (2)	H62A—C62—H62B	110.00
C42—C41—C46	111.4 (2)	C63—C62—H62B	109.00
C41—C42—C43	110.4 (3)	C62—C63—H63B	109.00
C42—C43—C44	111.9 (3)	H63A—C63—H63B	110.00
C43—C44—C45	110.7 (3)	C62—C63—H63A	109.00
C44—C45—C46	111.2 (3)	C64—C63—H63B	109.00
C41—C46—C45	110.6 (2)	C64—C63—H63A	109.00
N3—C31—H31	109.00	C65—C64—H64B	109.00
C36—C31—H31	109.00	C65—C64—H64A	109.00
C32—C31—H31	108.00	C63—C64—H64B	110.00
H32A—C32—H32B	109.00	C63—C64—H64A	109.00
C31—C32—H32A	109.00	H64A—C64—H64B	109.00
C33—C32—H32A	109.00	C66—C65—H65A	109.00
C33—C32—H32B	109.00	C64—C65—H65A	109.00
C31—C32—H32B	109.00	C64—C65—H65B	109.00
C34—C33—H33B	109.00	H65A—C65—H65B	109.00
C32—C33—H33A	109.00	C66—C65—H65B	109.00
C32—C33—H33B	109.00	C61—C66—H66B	110.00
H33A—C33—H33B	109.00	H66A—C66—H66B	109.00
C34—C33—H33A	109.00	C61—C66—H66A	109.00
H34A—C34—H34B	109.00	C65—C66—H66B	109.00
C33—C34—H34B	109.00	C65—C66—H66A	109.00
C35—C34—H34B	110.00	O11—C11—C12	116.1 (3)
C33—C34—H34A	109.00	O11—C11—O12	126.7 (3)
C35—C34—H34A	109.00	O12—C11—C12	117.1 (3)
C34—C35—H35A	109.00	C11—C12—C13	111.9 (2)
C36—C35—H35B	109.00	N11—C12—C11	112.8 (2)
C36—C35—H35A	109.00	N11—C12—C13	107.7 (2)
C34—C35—H35B	109.00	C12—C13—C14	116.4 (2)
H35A—C35—H35B	109.00	C13—C14—C15	121.3 (3)
C31—C36—H36B	109.00	C13—C14—C19	120.5 (3)
H36A—C36—H36B	110.00	C15—C14—C19	118.1 (3)
C35—C36—H36B	109.00	C14—C15—C16	121.2 (3)
C31—C36—H36A	109.00	C15—C16—C17	120.0 (3)
C35—C36—H36A	109.00	C16—C17—C18	119.7 (3)
C46—C41—H41	109.00	C17—C18—C19	120.7 (4)
N3—C41—H41	108.00	C14—C19—C18	120.4 (3)
C42—C41—H41	108.00	C13—C12—H12	108.00
C43—C42—H42A	109.00	N11—C12—H12	108.00
C41—C42—H42B	109.00	C11—C12—H12	108.00
H42A—C42—H42B	109.00	C14—C13—H13A	108.00
C43—C42—H42B	110.00	C14—C13—H13B	108.00
C41—C42—H42A	109.00	H13A—C13—H13B	110.00
C42—C43—H43A	109.00	C12—C13—H13A	108.00
H43A—C43—H43B	109.00	C12—C13—H13B	108.00
C42—C43—H43B	109.00	C14—C15—H15	119.00
C44—C43—H43B	109.00	C16—C15—H15	120.00
C44—C43—H43A	109.00	C15—C16—H16	120.00
C45—C44—H44A	110.00	C17—C16—H16	120.00
C43—C44—H44B	109.00	C18—C17—H17	120.00
C45—C44—H44B	109.00	C16—C17—H17	120.00
H44A—C44—H44B	110.00	C17—C18—H18	119.00
C43—C44—H44A	109.00	C19—C18—H18	120.00
C46—C45—H45A	109.00	C18—C19—H19	120.00
C46—C45—H45B	109.00	C14—C19—H19	120.00
H45A—C45—H45B	110.00	O21—C21—O22	126.8 (3)
C44—C45—H45B	109.00	O21—C21—C22	115.7 (3)
C44—C45—H45A	109.00	O22—C21—C22	117.6 (3)
C41—C46—H46A	109.00	N21—C22—C21	114.7 (2)
C41—C46—H46B	109.00	N21—C22—C23	112.9 (2)
C45—C46—H46A	110.00	C21—C22—C23	112.2 (3)
C45—C46—H46B	109.00	C22—C23—C24	117.1 (3)
H46A—C46—H46B	110.00	C23—C24—C25	119.6 (3)
N5—C51—C52	108.1 (2)	C23—C24—C29	122.6 (3)
C52—C51—C56	111.2 (2)	C25—C24—C29	117.7 (3)
N5—C51—C56	111.3 (2)	C24—C25—C26	119.7 (3)
C51—C52—C53	111.8 (2)	C25—C26—C27	120.5 (4)
C52—C53—C54	111.9 (3)	C26—C27—C28	120.7 (4)
C53—C54—C55	110.5 (3)	C27—C28—C29	119.2 (4)
C54—C55—C56	111.0 (3)	C24—C29—C28	122.1 (3)
C51—C56—C55	110.7 (3)	N21—C22—H22	105.00
N5—C61—C66	108.5 (2)	C21—C22—H22	106.00
N5—C61—C62	112.3 (2)	C23—C22—H22	106.00
C62—C61—C66	111.1 (2)	C22—C23—H23A	107.00
C61—C62—C63	110.1 (3)	C22—C23—H23B	107.00
C62—C63—C64	111.5 (3)	C24—C23—H23A	107.00
C63—C64—C65	111.2 (3)	C24—C23—H23B	107.00
C64—C65—C66	111.0 (3)	H23A—C23—H23B	110.00
C61—C66—C65	110.9 (3)	C24—C25—H25	120.00
C56—C51—H51	109.00	C26—C25—H25	120.00
C52—C51—H51	109.00	C25—C26—H26	119.00
N5—C51—H51	108.00	C27—C26—H26	120.00
C53—C52—H52A	109.00	C26—C27—H27	119.00
C51—C52—H52B	109.00	C28—C27—H27	120.00
C51—C52—H52A	109.00	C27—C28—H28	120.00
H52A—C52—H52B	109.00	C29—C28—H28	121.00
C53—C52—H52B	109.00	C24—C29—H29	119.00
C52—C53—H53B	108.00	C28—C29—H29	119.00
			
C41—N3—C31—C32	−178.6 (2)	C66—C61—C62—C63	56.8 (3)
C41—N3—C31—C36	−55.6 (3)	N5—C61—C62—C63	178.5 (2)
C31—N3—C41—C42	−55.0 (3)	C61—C62—C63—C64	−56.8 (4)
C31—N3—C41—C46	−178.4 (2)	C62—C63—C64—C65	56.1 (4)
C51—N5—C61—C66	−179.0 (2)	C63—C64—C65—C66	−55.2 (4)
C51—N5—C61—C62	57.8 (3)	C64—C65—C66—C61	55.2 (4)
C61—N5—C51—C56	55.8 (3)	O11—C11—C12—C13	−42.0 (4)
C61—N5—C51—C52	178.1 (2)	O11—C11—C12—N11	−163.7 (3)
N12—N11—C12—C13	171.8 (3)	O12—C11—C12—C13	140.6 (3)
N12—N11—C12—C11	−64.3 (4)	O12—C11—C12—N11	19.0 (4)
N22—N21—C22—C21	52.0 (4)	C11—C12—C13—C14	172.1 (3)
N22—N21—C22—C23	−78.2 (3)	N11—C12—C13—C14	−63.4 (4)
C32—C31—C36—C35	−55.0 (3)	C12—C13—C14—C15	−51.7 (4)
N3—C31—C36—C35	−175.3 (2)	C12—C13—C14—C19	132.8 (3)
N3—C31—C32—C33	174.8 (2)	C13—C14—C15—C16	−175.0 (3)
C36—C31—C32—C33	52.3 (3)	C19—C14—C15—C16	0.7 (5)
C31—C32—C33—C34	−52.4 (3)	C13—C14—C19—C18	175.8 (3)
C32—C33—C34—C35	55.2 (3)	C15—C14—C19—C18	0.1 (5)
C33—C34—C35—C36	−58.0 (3)	C14—C15—C16—C17	−0.8 (5)
C34—C35—C36—C31	58.1 (3)	C15—C16—C17—C18	0.1 (5)
N3—C41—C42—C43	−177.3 (2)	C16—C17—C18—C19	0.6 (6)
C42—C41—C46—C45	56.3 (3)	C17—C18—C19—C14	−0.8 (6)
N3—C41—C46—C45	−179.8 (2)	O21—C21—C22—N21	−179.8 (3)
C46—C41—C42—C43	−55.6 (3)	O22—C21—C22—N21	0.6 (4)
C41—C42—C43—C44	55.7 (4)	O22—C21—C22—C23	131.1 (3)
C42—C43—C44—C45	−56.0 (4)	O21—C21—C22—C23	−49.3 (4)
C43—C44—C45—C46	56.2 (4)	N21—C22—C23—C24	−57.7 (3)
C44—C45—C46—C41	−56.4 (3)	C21—C22—C23—C24	170.9 (2)
N5—C51—C56—C55	176.5 (2)	C22—C23—C24—C29	−33.7 (4)
C52—C51—C56—C55	56.0 (3)	C22—C23—C24—C25	150.0 (3)
C56—C51—C52—C53	−53.9 (3)	C23—C24—C25—C26	175.7 (3)
N5—C51—C52—C53	−176.3 (2)	C23—C24—C29—C28	−176.3 (3)
C51—C52—C53—C54	53.4 (3)	C25—C24—C29—C28	0.1 (5)
C52—C53—C54—C55	−54.8 (3)	C29—C24—C25—C26	−0.8 (5)
C53—C54—C55—C56	57.0 (3)	C24—C25—C26—C27	0.5 (6)
C54—C55—C56—C51	−58.0 (3)	C25—C26—C27—C28	0.6 (6)
N5—C61—C66—C65	179.4 (2)	C26—C27—C28—C29	−1.3 (6)
C62—C61—C66—C65	−56.7 (3)	C27—C28—C29—C24	1.0 (5)

Symmetry codes: (i) *x*+1, *y*, *z*+1; (ii) *x*−1, *y*, *z*; (iii) *x*, *y*, *z*+1; (iv) *x*−1, *y*, *z*−1; (v) *x*, *y*, *z*−1; (vi) *x*+1, *y*, *z*; (vii) *x*+1, *y*+1, *z*+1; (viii) *x*−1, *y*−1, *z*−1.

### Hydrogen-bond geometry (Å, º)

**Table:**

*D*—H···*A*	*D*—H	H···*A*	*D*···*A*	*D*—H···*A*
N3—H3*A*···O11	0.85	1.93	2.765 (3)	168
N3—H3*B*···O22	0.85	1.88	2.712 (3)	167
N5—H5*A*···O12	0.85	1.90	2.741 (3)	168
N5—H5*B*···O21	0.85	1.90	2.725 (3)	164
